# Network Toxicology and Machine Learning Uncover BPA-Driven Molecular Mechanisms in Atopic Dermatitis

**DOI:** 10.3390/cimb48070652

**Published:** 2026-06-25

**Authors:** Xingxin Cao, Xiangkai Cai, Mingxue Li, Weihua Jin, Fengmei Yang, Suqin Duan, Yanyan Li, Zhanlong He

**Affiliations:** Institute of Medical Biology, Chinese Academy of Medical Sciences & Peking Union Medical College, Kunming 650118, China; caoxingxin@student.pumc.edu.cn (X.C.); duansuqin@imbcams.com.cn (S.D.)

**Keywords:** atopic dermatitis, BPA, network toxicology, machine learning

## Abstract

Bisphenol A (BPA) is a common industrial chemical primarily used in the manufacture of plastics, and it has been found in more than 90% of people worldwide. As an endocrine disruptor, BPA can impair reproduction, development, immunity, metabolism, and cognition; it also disturbs immune balance and thus fosters chronic inflammation. A number of population-based studies have indicated a link between environmental BPA exposure and atopic dermatitis (AD). Nevertheless, the detailed molecular pathways connecting BPA to AD remain poorly understood. AD is the leading chronic recurrent inflammatory skin disorder, characterized by severe itching and repeated eczema-like lesions. Its prevalence is roughly 13% among children and 5% among adults, and its global incidence continues to rise, imposing heavy health and economic burdens on societies. To clarify whether and how BPA may promote or worsen AD, we carried out a comprehensive computational study that integrated network toxicology, transcriptomic data, machine learning, molecular docking, and molecular dynamics simulations. From the CTD, ChEMBL, and SwissTargetPrediction databases, we collected 5701 potential BPA targets; from GeneCards and OMIM, we obtained 3270 genes linked to AD. The overlap between these two gene sets gave a group of common candidate genes. Enrichment analyses using GO and KEGG showed that these common genes were significantly overrepresented in the PI3K-Akt signaling pathway, Th17 cell differentiation, and the JAK-STAT signaling pathway—all central to immune and inflammatory regulation. We then built a protein–protein interaction (PPI) network by submitting the common genes to the STRING database and employed Cytoscape to extract hub genes from that network. By integrating human AD transcriptomic profiles with the hub genes and applying two machine learning techniques (LASSO and SVM), we identified six core toxic targets of BPA in AD: *TIGIT*, *JAK3*, *IL22*, *S100A8*, *CCL2*, and *FCER1G*. These six targets fall into two main functional categories: immune dysregulation and inflammatory cell infiltration. Subsequent molecular docking and molecular dynamics simulation experiments confirmed that BPA binds well to all six targets and can form stable complexes with them. Collectively, our findings offer a preliminary experimental foundation for future investigations into the pathogenesis of BPA-induced AD and provide important molecular evidence for understanding how environment–gene interactions contribute to complex inflammatory skin diseases such as AD.

## 1. Introduction

Bisphenol A (BPA) is a widely used industrial chemical employed in the manufacture of plastics for canned food and beverage packaging, baby and water bottles, dental sealants, and many other consumer products [1]. Urinary biomonitoring studies detect BPA in over 90% of the population across multiple countries, indicating pervasive exposure [2]. Pregnancy is a physiologically distinct and vulnerable period during which exposure remains common; a study in a cohort of 832 mother–infant pairs detected BPA in the urine of 85% of pregnant women [3]. Although BPA has relatively low acute toxicity, it is a well-established endocrine disruptor that impairs reproductive, developmental, immune, metabolic, and cognitive functions and is considered a potential environmental contributor to several cancers [1]. Beyond endocrine disruption, BPA also perturbs immune function and provokes inflammatory responses [4]. In atopic dermatitis (AD), epidemiological evidence links high maternal BPA exposure during pregnancy with an increased risk of AD in infants and young children [5]. A clinical study demonstrated that BPA exposure was associated with hypomethylation of genes involved in the JAK-STAT and PI3K–AKT signaling pathways, including *IL33*, *TYK2*, *JAK1*, and *JAK3*, in the blood of pregnant women and that this epigenetic alteration may in turn impair skin barrier function and immune responses, suggesting that BPA exerts adverse effects on AD through epigenetic mechanisms [6].

AD is one of the most common chronic inflammatory skin disorders worldwide, affecting about 10% of people of all ages and continuing to increase in prevalence [7]. Its pathogenesis reflects a combination of impaired skin barrier function, immune dysregulation, and environmental exposures, which produce recurrent eczematous lesions, intense pruritus, sleep disturbance, and other symptoms that substantially reduce quality of life [8]. Environmental agents exert a strong influence on AD risk; for example, polycyclic aromatic hydrocarbons activate the aryl hydrocarbon receptor (AhR), induce cytokine and inflammatory mediator production, and thereby promote AD through multiple pathways [9]. These findings underscore the need to define how environmental exposures causally drive AD. Although epidemiological studies associate BPA exposure with AD, the molecular mechanisms—especially BPA’s principal toxic targets and the signaling pathways involved—remain unclear. Network toxicology, which integrates network pharmacology and systems biology, constructs compound–target–disease association networks and, when combined with big data analytics and multi-omics, offers a rigorous framework for dissecting complex toxicological mechanisms [10]. Integrating transcriptomic data with machine learning refines these networks to highlight likely core toxic targets [11]. Molecular docking and molecular dynamics simulations then evaluate binding affinity and complex stability to characterize how BPA interacts with candidate core targets [12].

Although both epidemiological and in vivo studies have implicated BPA in the pathogenesis of AD, the specific molecular targets and signaling pathways mediating BPA-induced AD remain poorly understood, both experimentally and computationally. To address this knowledge gap, we developed an integrative computational framework combining network toxicology, transcriptomics profiling, and machine learning algorithms to systematically prioritize candidate targets. The binding affinity and complex stability between BPA and these prioritized targets were further validated via molecular docking and molecular dynamics simulations. This multi-tiered in silico strategy was designed to identify core toxicological targets underlying BPA-associated AD, to elucidate the key inflammatory and pruritic signaling pathways perturbed by BPA, and to provide a computational foundation and preliminary reference for guiding future experimental validation and environmental risk assessment of BPA-related AD.

## 2. Materials and Methods

### 2.1. Identification of Bisphenol A Targets in Atopic Dermatitis

Human genes affected by BPA were retrieved from SwissTargetPrediction (https://swisstargetprediction.ch/, accessed on 31 December 2025), ChEMBL (https://www.ebi.ac.uk/chembl/, accessed on 8 December 2025), and CTD (https://ctdbase.org/, accessed on 31 December 2025). For SwissTargetPrediction, the 2D SDF of BPA (CID: 6623), downloaded from PubChem (https://pubchem.ncbi.nlm.nih.gov/, accessed on 8 December 2025), was uploaded; the species was set to “Homo sapiens”, and the probability threshold was set above 0. In ChEMBL and CTD, queries employed the term “bisphenol A” with the species restricted to “Homo sapiens”. Genes associated with AD were obtained from GeneCards (https://www.genecards.org/, accessed on 8 December 2025) and Online Mendelian Inheritance in Man (OMIM; https://www.omim.org/, accessed on 31 December 2025) using the search term “atopic dermatitis”. Potential toxicity targets of BPA for AD were identified by intersecting the BPA-affected genes with the AD-related genes using the bioinformatics platform provided by Bioinformatics.com.cn (https://www.bioinformatics.com.cn/, accessed on 31 December 2025).

### 2.2. Protein–Protein Interaction Network Construction and Hub-Gene Identification

The PPI network for BPA in AD was constructed by uploading the toxicological genes identified in Section 2.1 to STRING (https://cn.string-db.org/, accessed on 31 December 2025) with the organism set to “Homo sapiens” and exporting the results as tab-separated values (TSV). Hub genes are defined as nodes with high centrality within a PPI network and are considered to play critical roles in both network topology and biological function. To identify hub genes, Degree, Betweenness, and Closeness centrality indices were calculated from the TSV file using the CentiScaPe 2.2 plugin within Cytoscape 3.10.3 (Cytoscape Consortium, La Jolla, San Diego, CA, USA). The Degree value of a node denotes the total number of directly connected nodes in the network; a higher Degree value indicates greater significance for the node. Closeness is the reciprocal of the sum of the shortest path lengths from the node to all other nodes in the network; a higher Closeness value implies that the node is centrally located in the network and can swiftly influence the entire system. Betweenness measures the fraction of the shortest paths between all pairs of nodes that pass through this node; nodes with high Betweenness values act as “bottlenecks,” controlling the flow of information or signals. Nodes exhibiting high values across all three metrics were identified as central hubs, which are considered to exert strong regulatory influence on network stability and function. The hub-gene interaction network was then visualized in Cytoscape 3.10.3.

### 2.3. Functional Enrichment Analysis of Hub Genes

Gene Ontology (GO) and Kyoto Encyclopedia of Genes and Genomes (KEGG) enrichment analyses were performed by submitting the hub genes to the Database for Annotation, Visualization, and Integrated Discovery (DAVID; https://davidbioinformatics.nih.gov/, accessed on 31 December 2025). Significance thresholds were set at *p* < 0.01 and FDR < 0.01. The 20 most significant GO terms within each category—biological process (BP), cellular component (CC), and molecular function—and the top 20 KEGG pathway terms ranked by ascending *p*-value were visualized using the bioinformatics platform provided by Bioinformatics.com.cn, as described in Section 2.1.

### 2.4. Machine Learning-Based Identification of Core Toxicity Targets

Human transcript data for AD (GSE280220) were obtained from the Gene Expression Omnibus (GEO; https://www.ncbi.nlm.nih.gov/geo/, accessed on 31 December 2025). Genes with significant changes were defined by a fold change of <0.5 or >2 and a *t*-test *p*-value < 0.05 between healthy individuals and AD patients. Potential core toxicity targets were identified by overlapping these differentially expressed genes with hub genes. To refine the core toxicity gene set, we applied two machine learning methods: Least Absolute Shrinkage and Selection Operator (LASSO) regression and Support Vector Machine (SVM). LASSO, a linear regression technique with L1 regularization, was used for feature selection because it can shrink some regression coefficients to zero. We implemented LASSO with the glmnet package in R 4.4.3, using L1 regularization (alpha = 1), and selected the optimal lambda (lambda.min) by 10-fold cross-validation. The input comprised the centralized and standardized gene expression matrix, and the response variable was a binary classification (0 = normal, 1 = AD). The final model selected key features as those variables with nonzero coefficients at the lambda value that minimized cross-validation error. SVM is a supervised algorithm for classification and regression that finds the optimal hyperplane in feature space to separate classes and maximize the margin. SVM was carried out with the caret package using a linear kernel (svmLinear), 5-fold cross-validation, and feature selection over sequence sizes = seq(1, 23, by = 2). Data were standardized prior to model fitting, and the final model was trained with svmLinear2 using the default cost parameters. Core toxicity targets were defined as the intersection of the LASSO and SVM results.

### 2.5. Molecular Docking of Bisphenol A to Core Toxicity Targets

The energy-minimized structure of BPA was generated using Chem 3D (CambridgeSoft Corporation, Cambridge, MA, USA) from the SDF format file in Section 2.1 and saved as a MOL2 format file. The protein structures of the core toxicity targets were obtained from the RCSB Protein Data Bank (RCSB PDB; https://www.rcsb.org/, accessed on 31 December 2025), and water and organics were removed using PyMOL 3.0 (Schrödinger, South San Francisco, CA, USA). Molecular docking involved importing the BPA MOL2 format file and each core toxicity target’s PDB format file, adding hydrogens to the core toxicity targets, setting a grid box in AutoDock Tools 1.5.7 (Scripps Research, La Jolla, San Diego, CA, USA), and conducting molecular docking in AutoDock Vina 1.1.2 (Scripps Research, La Jolla, San Diego, CA, USA). The hydrogen bonds between BPA and the core toxicity targets in the molecular docking results were identified using Protein–Ligand Interaction Profiler (PLIP; https://plip-tool.biotec.tu-dresden.de/, accessed on 31 December 2025), and the docking interactions were visualized in PyMOL 3.0.

### 2.6. Molecular Dynamics Simulations of Bisphenol A–Target Complexes

The MOL2 format file of BPA was converted to a PDB format file using PyMOL 3.0. Subsequently, the PDB format files of BPA and core toxicity targets from Section 2.5 were imported into Yet Another Scientific Artificial Reality Application 10.3.16 (YASARA; YASARA Biosciences GmbH, Vienna, Austria) for molecular docking to determine the lowest binding energy and stereochemically reasonable geometry for subsequent molecular dynamics simulation. Prior to molecular docking in YASARA 10.3.16, water and other organic molecules were removed, the pH was adjusted to 7.4, and a cubic simulation box with periodic boundary conditions in “around-all-atoms” mode and a 5 Å buffer (α = β = γ = 90°) was established. The molecular complex exhibiting the most favorable binding energy was chosen for the molecular dynamics simulation. The 100 ns simulation utilized the same cubic simulation box as in molecular docking, employed the AMBER14 force field, specified the ion concentration as a mass fraction with 0.9% NaCl (physiological solution) ions (NaCl 0.9), and maintained a temperature of 298 K. The stability of the molecular dynamics simulation was assessed using root-mean-square deviation (RMSD), binding energy, and root-mean-square fluctuation (RMSF), and the results were visualized in GraphPad Prism 10.4.2 (GraphPad Software, San Diego, CA, USA).

## 3. Results

### 3.1. Bisphenol A Targets Associated with Atopic Dermatitis

**Figure 1:**
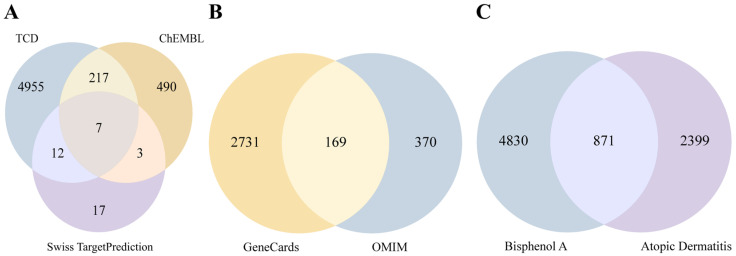
Identification of bisphenol A and atopic dermatitis targets: (**A**) Targets influenced by BPA were obtained from SwissTargetPrediction, ChEMBL, and CTD. (**B**) AD-related targets were retrieved from OMIM and GeneCards. (**C**) Common genes implicated in BPA-induced AD were determined by intersecting the BPA targets with the AD-related targets.

Targets affected by BPA were collected from SwissTargetPrediction (39), ChEMBL (517), and CTD (5191), yielding 5701 unique genes after removing duplicates (Figure 1A). AD-related targets were retrieved from OMIM (539) and GeneCards (2540), producing 3270 genes after deduplication (Figure 1B). The overlap between BPA and AD targets comprised 871 common genes, which were designated as toxicity targets mediating BPA-associated onset and progression of AD (Figure 1C).

### 3.2. Topology of the Protein–Protein Interaction Network and Hub Genes

**Figure 2:**
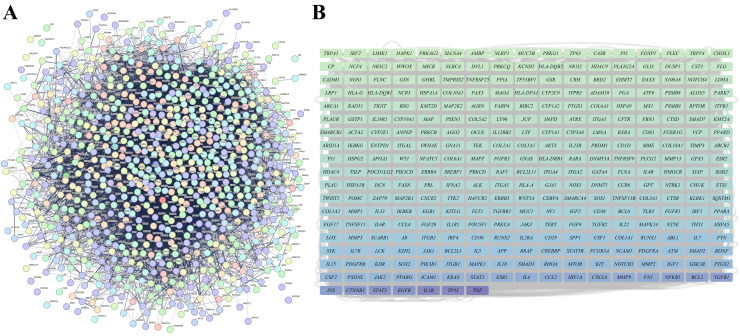
Protein–protein interaction network construction and hub-gene identification: (**A**) Protein–protein interaction (PPI) network of common genes implicated in BPA-associated initiation and progression of AD. (**B**) Hub genes identified by analysis with CentiScaPe 2.2 in Cytoscape 3.10.3 after importing the PPI file.

The protein–protein interaction (PPI) network of toxicity targets was established using STRING. Excluding isolated proteins, this network consists of 851 nodes and 15,483 edges, yielding an average node degree of 36.4 (Figure 2A). Hub genes were identified by selecting the top 50% of nodes based on Degree, Betweenness, and Closeness metrics computed by the analysis in CentiScaPe 2.2 in Cytoscape 3.10.3 after importing the PPI file [13]. Degree centrality reflects the number of direct connections a node maintains within the network. Closeness centrality captures the speed with which a node can disseminate signals to, or exert influence over, the entire network. Betweenness centrality denotes the extent to which a node serves as an intermediary, controlling or mediating the flow of information between other nodes. A total of 313 targets were recognized as hub genes, collectively displaying 18,626 edges. In the hub-gene network, nodes are color-coded from purple to blue to green to indicate decreasing Degree values (Figure 2B).

### 3.3. GO and KEGG Enrichment Analysis of the Identified Hub Genes

**Figure 3:**
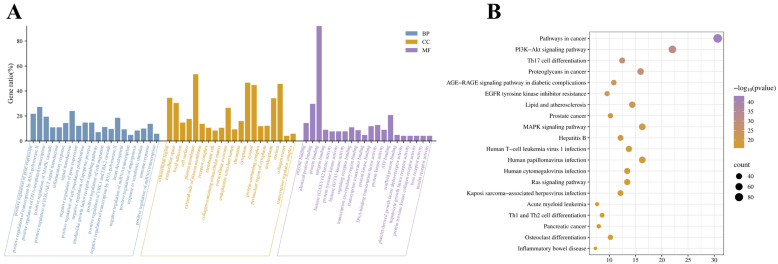
Functional enrichment analysis of common genes: (**A**) Integrated GO ternary bar showing the 20 most significant GO terms in each category: biological process, cellular component, and molecular function. (**B**) KEGG pathway enrichment dot-bubble plot for the top 20 entries.

For the pathological process of BPA-triggered AD, GO, and KEGG enrichment analyses were performed by submitting the hub genes to DAVID, and results were filtered using thresholds of *p* < 0.01 and FDR < 0.01. The 20 most significant GO terms in each category—BP, CC, and MF—are displayed in an integrated GO ternary bar (Figure 3A). The principal BPs included positive regulation of gene expression, positive regulation of PI3K-Akt signal transduction, inflammatory response, and immune response (Figure 3A). The predominant CCs involved the extracellular region, extracellular space, focal adhesion, cell surface, and plasma membrane. The major MFs comprised enzyme binding, identical protein binding, protein binding, integrin binding, and histone H2AXY142 kinase activity. KEGG pathway enrichment analysis revealed that the most significantly enriched signaling pathways included pathways in cancer, the PI3K-Akt signaling pathway, Th17 cell differentiation, the JAK-STAT signaling pathway, and Th1 and Th2 cell differentiation, with the top 20 enriched pathways presented in Figure 3B.

### 3.4. Core Toxicity Targets Identified by Machine Learning

**Figure 4:**
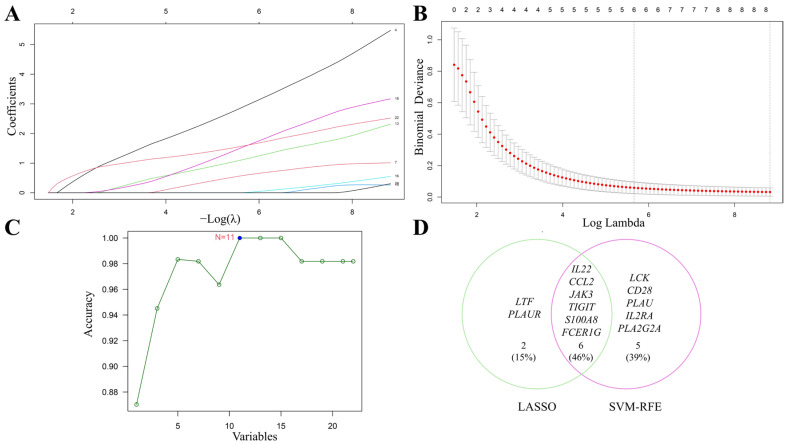
Identification of core toxicity targets using two machine learning algorithms: (**A**) Coefficient pathways from Least Absolute Shrinkage and Selection Operator (LASSO) regression. (**B**) LASSO filter results, with the optimal λ yielding 8 genes. (**C**) Support Vector Machine (SVM) results, which achieved maximum accuracy with 11 genes. (**D**) Intersection of LASSO and SVM outputs revealed 6 core toxicity genes associated with BPA-induced AD.

**Figure 5:**
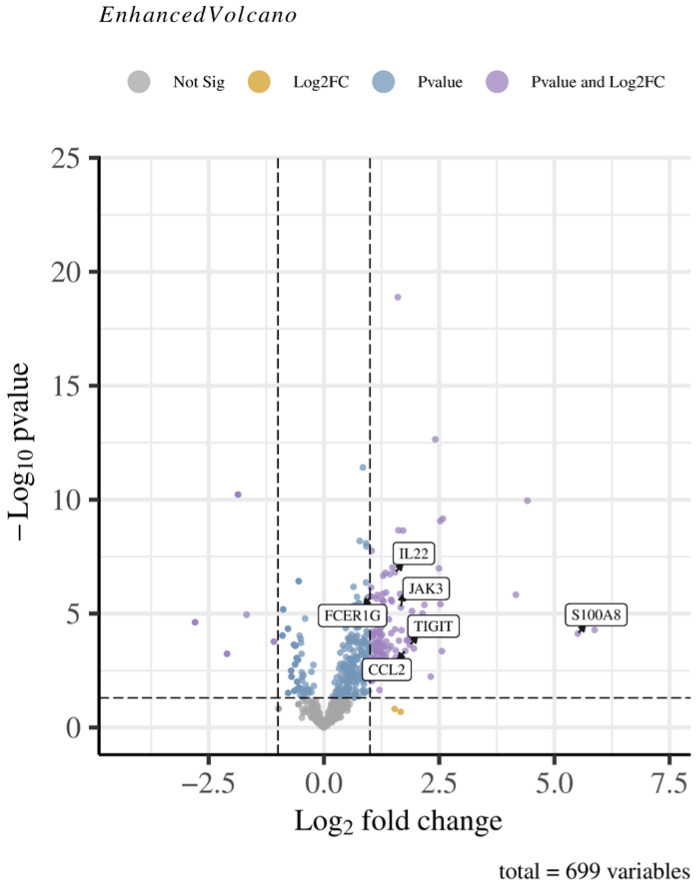
Expression profiles of six core toxicity targets in the transcriptome.

Human transcriptomic data for AD were obtained from GEO (GSE280220) [14,15]. Core toxicity targets were identified by integrating differentially expressed genes from this transcriptomic dataset with hub genes selected by two machine learning algorithms: LASSO regression and SVM. The LASSO coefficient profile plot from the LASSO regression illustrated each gene’s contribution to BPA-triggered AD pathology, with the most influential genes remaining nonzero later in the regularization path (*FCER1G* (4), *PLAUR* (7), *IL22* (13), *JAK3* (15), *CCL2* (16), *TIGIT* (18), *LTF* (20), and *S100A8* (22)) (Figure 4A). Binomial deviance assessed the LASSO model’s goodness of fit, and the minimum deviance occurred at the log lambda that selected 8 genes (Figure 4B). The SVM model achieved its highest accuracy when it produced 11 genes (Figure 4C). Intersection of the LASSO and SVM outputs yielded six core toxicity targets: *TIGIT*, *JAK3*, *IL22*, *S100A8*, *CCL2*, and *FCER1G* (Figure 4D). All six genes were significantly upregulated in the AD transcriptomic data (Figure 5).

### 3.5. High-Affinity Binding Modes of Bisphenol A to Core Toxicity Targets

**Figure 6:**
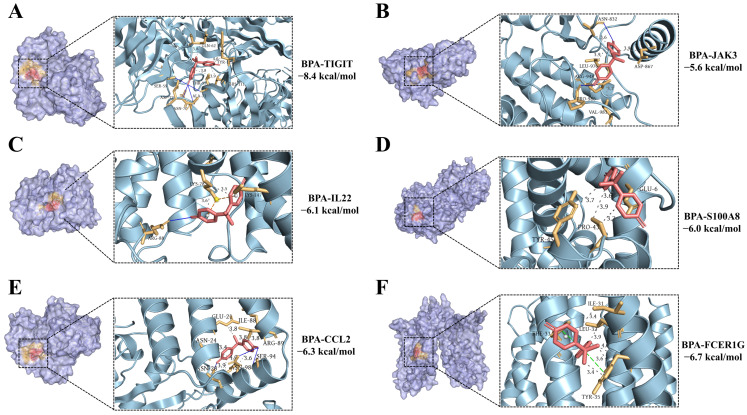
Molecular docking poses and binding energies of BPA with six core toxicity targets: (**A**) BPA–FCER1G (7Q5T). (**B**) BPA–IL22 (1M4R). (**C**) BPA–JAK3 (6DB3). (**D**) BPA–CCL2 (6CTW). (**E**) BPA–TIGIT (7VYT). (**F**) BPA–S100A8 (5HLV).

In silico docking of BPA with the core toxicity targets was carried out using AutoDock Vina 1.1.2. A complex of ligand and receptor was deemed stable if the energy of binding was below −5 kcal/mol [16]. Three-dimensional structures for six target proteins were obtained from the RCSB PDB: TIGIT (PDB ID: 7VYT), JAK3 (PDB ID: 6DB3), IL22 (PDB ID: 1M4R), S100A8 (PDB ID: 5HLV), CCL2 (PDB ID: 6CTW), and FCER1G (PDB ID: 7Q5T). All six BPA–protein complexes met the stability threshold, and hydrogen bonds plus interacting amino acid residues were annotated and visualized in PyMOL 3.0 (Figure 6).

### 3.6. Stability and Interaction Dynamics of Bisphenol A–Protein Complexes

**Figure 7:**
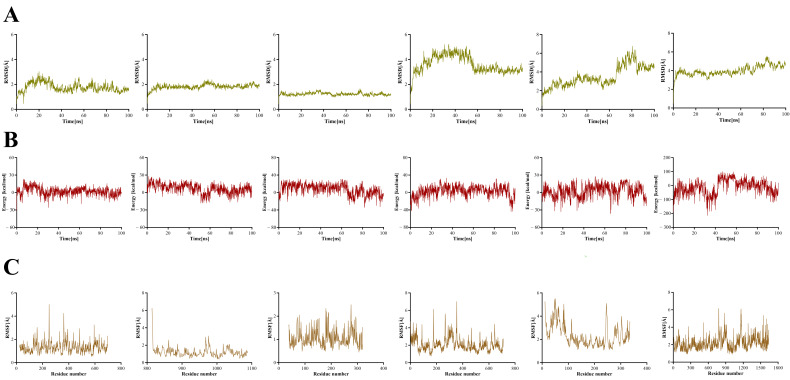
Molecular dynamics simulation of BPA with six core toxicity targets: (**A**) Root-mean-square deviation (RMSD) results, shown left to right for BPA bound to TIGIT, JAK3, IL22, S100A8, CCL2, and FCER1G. (**B**) Binding energy results, shown left to right for BPA bound to TIGIT, JAK3, IL22, S100A8, CCL2, and FCER1G. (**C**) Root-mean-square fluctuation (RMSF) results, shown left to right for BPA bound to TIGIT, JAK3, IL22, S100A8, CCL2, and FCER1G.

Molecular dynamics simulations of all six BPA–protein complexes were carried out with YASARA 10.3.16, and results were assessed using three metrics: RMSD, binding energy, and RMSF. RMSD measures the time-averaged deviation of the protein backbone from its initial conformation. Binding energy reflects the thermodynamic favorability of the interaction. RMSD and fluctuations in binding energy reflect the binding state of complexes under simulated human conditions; lower RMSD values and smaller binding-energy fluctuations indicate greater stability of the protein–ligand complex. RMSF quantifies the displacement of each atom or amino acid residue from its mean position during the simulation; larger RMSF values signify increased motion and greater flexibility of that atom or residue. Molecular dynamics simulations show that BPA remained stably bound to all six core toxicity targets. The RMSD values for the BPA–TIGIT, BPA–JAK3, and BPA–IL22 complexes largely stayed within 2 Å, while those for the BPA–S100A8, BPA–CCL2, and BPA–FCER1G complexes remained mostly within 4 Å (Figure 7A). The computed binding energies of BPA to these six targets were relatively stable and exhibited only modest fluctuations (Figure 7B). Except at the N- and C-termini, residues at all BPA–core toxicity protein binding sites displayed relatively low RMSF values (Figure 7C).

## 4. Discussion

AD is the predominant chronic, relapsing inflammatory skin disease, characterized by intense pruritus and repeated eczematous lesions, affecting approximately 13% of children and 5% of adults and showing an escalating worldwide occurrence that poses substantial health and socioeconomic burdens [17,18,19]. Although genetic factors such as FLG mutations primarily predispose susceptibility to AD, environmental triggers are increasingly implicated in its pathogenesis, notably the widespread endocrine disruptor BPA [20,21]. Epidemiological data associate prenatal or childhood BPA exposure with both the onset and exacerbation of AD in children, yet the molecular mechanisms mediating this link remain incompletely understood [22]. This study was the first to apply an integrated strategy—combining network toxicology, transcriptomics, machine learning, and molecular simulation—to delineate the molecular mechanism of BPA-induced AD. Using this approach, we identified six core toxicity targets, namely *S100A8*, *TIGIT*, *FCER1G*, *IL22*, *JAK3*, and *CCL2*, and highlighted key signaling pathways, including PI3K-Akt, Th17 cell differentiation, and JAK-STAT. These findings provide a methodological reference for studying how environmental chemicals induce complex inflammatory skin diseases at the systems-biology level and offer new scientific perspectives and potential targets for preventing environmental etiologies and for targeted interventions in AD.

The six core toxicity targets identified in this study suggest that BPA may promote AD through two principal modules: immune dysregulation (*TIGIT*/*IL-22*/*S100A8*/*FCER1*) and inflammatory cellular infiltration (*JAK3*/*CCL2*/*FCER1*). TIGIT is an inhibitory immune receptor that is predominantly expressed on regulatory T cells (Treg) and on exhausted T cells, where it supports immune tolerance and tissue repair [23]. TIGIT expression can rise as a compensatory response in AD patients, while BPA, a prototypical environmental endocrine disruptor, may impair TIGIT-mediated immunosuppression through epigenetic modification, disruption of downstream signaling, or related mechanisms [24,25]. Functionally intact TIGIT+ Treg cells suppress activation and proliferation of pathogenic Th22 cells, and TIGIT dysfunction can weaken that suppression, permitting excessive IL-22 production [26]. IL-22, produced primarily by activated Th22 cells, induces keratinocytes to overexpress antimicrobial proteins such as S100A8, S100A9, BD2, and BD3, and it inhibits terminal keratinocyte differentiation while increasing cellular motility; these combined effects compromise the skin barrier and exacerbate inflammation [27]. S100A8, a damage-associated molecular pattern (DAMP), amplifies cutaneous inflammation in AD; elevated S100A8 levels chemoattract neutrophils and suppress genes involved in keratinocyte differentiation, exacerbating barrier defects and perpetuating an “inflammation–barrier disruption” cycle [28]. JAK3, a member of the JAK-STAT family, transduces signals from Th2 cytokines such as IL-4 and IL-13, which drive chronic inflammation in AD [29,30]. Our enrichment analyses and molecular modeling support the hypothesis that BPA may activate the JAK-STAT pathway. Prenatal bisphenol exposure has been associated with hypomethylation of JAK-STAT pathway genes—key regulators of skin barrier and immune function—suggesting a potential link to AD [6]. BPA or its metabolites could alter JAK3 phosphorylation and thereby sustain STAT activation, either via oxidative stress or by direct interaction, leading to transcriptional induction of inflammatory and pruritus-related genes [31]. Excessive IL-4 and IL-13 downstream of JAK3 can activate sensory neurons through the shared receptor subunit IL-4Rα and Janus kinase 1 to provoke chronic itch while also promoting persistent upregulation of CCL2 [32]. Elevated CCL2 recruits monocytes, macrophages, and other immune cells, amplifies Th2-type inflammation and barrier disruption, and establishes a self-reinforcing inflammatory microenvironment that maintains chronic skin inflammation [33]. The combined disruptive effects of these proteins and cytokines on the skin barrier facilitate allergen and microbe penetration, thereby persistently activating FCER1G-dependent immune responses [34]. This activation promotes degranulation and the release of histamine, leukotrienes, and other inflammatory mediators, which directly provoke acute pruritus and inflammation in AD [35]. Molecular docking and molecular dynamics simulations confirmed BPA interactions with these six targets and demonstrated favorable binding properties, providing computational support for a mechanistic basis of BPA-induced AD.

KEGG enrichment of the common genes connected diverse targets into pathway networks with clear biological relevance, clarifying the multilayered mechanisms by which BPA induces AD. Strong enrichment of “Th17 cell differentiation” and the “JAK-STAT signaling pathway” underscores a pronounced effect of BPA on adaptive immunity. Th17 cells and their effector IL-17A are central to intrinsic AD; prior studies suggest that BPA may promote Th17 differentiation by modulating RORγt expression, and this study is the first to evaluate that effect specifically in the context of AD [36,37]. The “PI3K-Akt signaling pathway” and the “MAPK signaling pathway” regulate cell proliferation, survival, and stress responses [38]. BPA-induced oxidative stress can activate these pathways, which may promote abnormal keratinocyte survival and amplify inflammatory responses via downstream transcription factors [39,40]. This likely marks a critical nexus between environmental stressors and cutaneous inflammatory pathology. The enrichment of “pathways in cancer” is notable because it points to molecular mechanisms shared by chronic inflammation and tumorigenesis—sustained proliferative signaling, evasion of apoptosis, and a persistent inflammatory microenvironment. Collectively, these results indicate that prolonged BPA exposure may raise the risk of pathological skin changes by sustaining or worsening chronic skin inflammation. The findings highlight the tissue specificity and disease-context dependence of BPA’s toxic effects in triggering AD. In susceptible individuals, BPA may preferentially elicit systemic autoimmunity or organ-specific inflammatory responses that initiate AD, while progression to clinical disease depends on genetic background, exposure windows, and co-exposures.

To our knowledge, this study is the first to apply an integrated computational strategy to systematically map the molecular network through which BPA contributes to AD, thereby linking an environmental exposure to specific cutaneous immune–pathological processes. The core targets we identified provide a theoretical foundation for developing agents that selectively block BPA’s toxic effects or for screening safer BPA alternatives. The study also faces inevitable limitations. Computational simulations generate strong mechanistic hypotheses, but the predicted interactions require functional validation in vivo and in vitro. For example, these findings could be validated and extended by treating human keratinocyte cell lines with BPA or by inducing AD in mice through controlled BPA exposure. Second, this study relies mainly on databases that reflect static or acute exposures and therefore does not capture the complex effects of chronic, low-dose, mixed exposures that occur in real life. How interindividual genetic variation alters sensitivity to BPA toxicity also remains a critical question for precision environmental medicine. Additionally, exposomic studies nested within prospective birth cohorts and incorporating epigenetic analyses could clarify early-life programming mechanisms through which BPA exposure influences AD development. Finally, assessing combined exposures of BPA with other common environmental toxicants, such as phthalates and PM2.5, would provide a more realistic evaluation of environmental risk. Our study provides essential preliminary data to support future research on BPA-induced AD and presents novel molecular evidence connecting “environment–gene” interactions to complex diseases. This emphasizes the urgent necessity to enhance public health interventions for controlling endocrine-disrupting environmental pollutants and protecting vulnerable populations, particularly children. The amalgamation of predictive capabilities in computational toxicology with the meticulous validation of experimental research will serve as a potent tool in unraveling the origins of environmentally mediated disorders and promoting preventive healthcare.

## 5. Conclusions

Our study found that BPA promotes AD via a network pathogenic axis centered on six core toxicological targets: *TIGIT*, *JAK3*, *IL22*, *S100A8*, *CCL2*, and *FCER1G*. These targets belonged to two principal modules, namely immune dysregulation (*TIGIT*/*IL22*/*S100A8*/*FCER1G*) and inflammatory cellular infiltration (*JAK3*/*CCL2*/*FCER1G*), which collectively sustain chronic cutaneous inflammation and itch. BPA also perturbs multiple signaling pathways, notably PI3K–Akt, Th17 differentiation, and JAK–STAT. Through these mechanisms, BPA disrupts cutaneous immune homeostasis and sustains a proinflammatory milieu that contributes to AD.

From a translational perspective, these six targets are candidate biomarkers for BPA-associated AD risk and potential therapeutic nodes. Our integrative computational framework—merging network toxicology, transcriptomics, machine learning, and molecular simulation—offers a systems-level approach for in silico investigation of disease mechanisms triggered by environmental pollutants. While these predictions require rigorous experimental validation, and future studies should evaluate chronic low-dose co-exposure with other endocrine disruptors, this work provides a mechanistic foundation for understanding environment–gene interactions in AD and highlights the urgent need for BPA exposure reduction strategies, particularly for pregnant women and children.

## Data Availability

The original contributions presented in this study are included in the article. Further inquiries can be directed to the corresponding author.

## Abbreviation

The following abbreviation are used in this manuscript.

**Abbreviation**	**Full Term**
BPA	bisphenol a
AD	atopic dermatitis
AhR	aryl hydrocarbon receptor
ChEMBL	Chemical Biology and Medicinal Chemistry Database
CTD	Comparative Toxicogenomics Database
OMIM	Online Mendelian Inheritance in Man
RCSB PDB	RCSB Protein Data Bank
TSV	tab-separated values
GO	Gene Ontology
KEGG	Kyoto Encyclopedia of Genes and Genomes
BP	biological process
CC	cellular component
MF	molecular function
LASSO	Least Absolute Shrinkage and Selection Operator
SVM	Support Vector Machine
YASARA	Yet Another Scientific Artificial Reality Application
PPI	protein–protein interaction
DAVID	The Database for Annotation, Visualization, and Integrated Discovery
RMSD	root-mean-square deviation
RMSF	root-mean-square fluctuation
